# Ontario Veterinary College

**Published:** 1896-09

**Authors:** 


### AMONG THE COLLEGES.

### ONTARIO VETERINARY COLLEGE.

THE opening lecture of the session of 1896–97 will occur on Wednesday, October 14th. No special changes are announced in connection with this school. The same corps of instructors will be maintained. During the period between sessions Prof. J. T. Duncan, of the faculty, has been abroad with a view of gaining a wider knowledge of the best methods of teaching extant. The schpol looks for a greater attendance this year than last, as its facilities for teaching were never so complete as at present.

